# Yellow Fever—More a Policy and Planning Problem than a Biological One

**DOI:** 10.3201/eid2210.160875

**Published:** 2016-10

**Authors:** Charles H. Calisher, John P. Woodall

**Affiliations:** Colorado State University, Fort Collins, Colorado, USA (C.H. Calisher);; Program for Monitoring Emerging Diseases, Brookline, Massachusetts, USA (J.P. Woodall)

**Keywords:** yellow fever, yellow fever viruses, viruses, yellow fever vaccine, outbreak, epidemic, vaccination, arbovirus, Aedes aegypti, mosquitoes, epidemiology, policy and planning, dose-sparing, Angola, Asia, China

**To the Editor:** The recent and ongoing outbreak (epidemic) of yellow fever (YF) in Angola is cause for concern, not only in West Africa, but also in contiguous and other nearby countries (*1*). As of June 21, 2016, the World Health Organization (WHO) had reported 3,137 cases (847 laboratory-confirmed) and 350 deaths (*2*), but at the present time, it is not cause for panic or for extravagant claims of an “impending global health threat” (*3*). As long as the Angola Ministry of Health reports that there have been <400 deaths from YF since it declared the outbreak 4 months ago, and because there is an effective vaccine against this disease, it is difficult to understand dire warnings of a global threat.

WHO considers the situation of high concern because of the inadequate surveillance system in Angola, one that is incapable of identifying new foci or areas where cases might emerge (*2*). Such an inability suggests that many more cases and deaths have already occurred (*4*). What is needed now, without further unconscionable delays, is a proper vaccination campaign, one that has been unachievable to now. We here reaffirm what has been suggested by the WHO Strategic Advisory Group of Experts on Immunization and by others, but as of June 20, 2016, not applied by WHO: that the YF vaccine administered at one fifth of the regular dose could be used until the epidemic ends (*5*).

Despite vaccination campaigns in various provinces in Angola, circulation of YF virus (YFV; family *Flaviviridae*, genus *Flavivirus*) in some districts persists. Attempts to control this epidemic are being made by application of the effective YFV 17D vaccine that has been used for many decades worldwide. Whereas recognition of cases of YF has decreased in Angola, cases continue to occur there, and isolated cases have been detected in persons who have visited Angola as tourists or for business purposes. Furthermore, cases in nearby Democratic Republic of the Congo have increased. Because YF is not endemic to Asia, such patients have the potential to serve as primary sources of YFV and as index sources for subsequent clusters, outbreaks, or epidemics, not only in China, but elsewhere in Asia, which is a nightmare scenario.

The principal mosquito vector of YFV is *Aedes aegypti*, which is found in southern China and elsewhere in Asia, as are *Ae. albopictus* mosquitoes (*6*), which can also transmit YFV and serve as a bridging vector between jungle and urban cycles of YFV in a variety of ecosystems (*7*). These mosquitoes feed on humans and are found peridomestically. Mosquitoes of these species also are capable of transmitting dengue viruses, chikungunya virus, Zika virus, and other human pathogens. Their presence should serve as a warning to local health authorities of potential arbovirus disease outbreaks and, therefore, to maintain or initiate mosquito vector control programs. Most industrialized countries are aware of these warnings; the 40 YF-endemic countries, predominantly tropical areas in Africa and Central and South America (≈90% of cases reported every year occur in sub-Saharan Africa), maintain diagnostic competence and surveillance systems, including clinical findings, testing of sick nonhuman primates and arthropods, and other indicators. Four countries that produce YF vaccine have purchased stocks or have arrangements in place to obtain sufficient doses in instances of immediate need.

Because destinations of an increasing number of travelers include YF-endemic areas, national and international regulations require a recent (<10 years) verified history of vaccination against this virus; China does not have such regulations. If a person traveling to a recognized YF-endemic area is not required to be vaccinated in advanced, then they are essentially on their own with regard to self-protection, but the greater threat is to their own country, if and when they return.

Of ostensibly great concern has been 11 unvaccinated YF-infected Chinese residents and workers who returned from Angola to China; others probably returned elsewhere, including to the Democratic Republic of the Congo, where 1,400 suspected cases (53 imported from Angola), including 82 deaths, have occurred, including some secondary cases, and to Kenya (*1*). Several YF cases have been detected recently in Uganda but are not related to the Angola outbreak. In China, all recognized YF patients have been isolated and given appropriate medical care; secondary cases have not been detected. Thus, all cases of YF in China were imported. Nonetheless, should secondary cases begin to be detected in areas in Asia to which *Ae. aegypti* or *Ae. albopictus* mosquitoes are endemic, a door would open for more widespread extension of YFV and for establishment of enzootic and endemic situations, which are unheard of in most parts of the temperate world. The last thing China in particular and Asia in general need is to become endemic for YF.

The question “why has there been no YF in Asia?” has been asked for decades, even before global climate change was recognized, and various hypotheses have been put forward. *Ae. aegypti* mosquitoes from various geographic sources and with corresponding genetic variations at different isozyme loci were infected orally with YF virus (*8*). Subsequent infection rates suggested that *Ae. aegypti* mosquito populations with genetic similarities had similar rates of infection with the virus. Tabachnick et al. concluded that there are differences between New World and Asian mosquito populations in this regard (*8*). Whether such differences might account for preclusion of YFV from Asia has not been proven. Antibodies against dengue viruses, which are widely present in Southeast Asia, might be protective against YFV infections, but studies by Izurieta et al. (*9*), Weiland et al. (*10*), and Agampodi and Wickramage (*11*) suggest otherwise. Because YFV is a zoonotic agent whose natural cycle involves nonhuman primates and mosquitoes other than *Ae. aegypti*, it is not possible to eradicate these mosquitoes by using available tools; thus, we rely on application of YF vaccine to protect humans.

Countries in YF-endemic areas or with a history of YF and that do not have adequate resources to enable them to set aside funds for controlling diseases, or that do not request assistance in formulating expanded childhood immunization programs, or that spend the funds they have available on other projects must be clearly informed that they are potential dangers to the rest of us. Obviously, not all countries need to vaccinate their residents against all viruses for which vaccines are available. However, countries not vaccinating when there are potential risks might seriously consider reevaluating their requirements for persons traveling to or returning from YF-endemic areas and for protecting key population groups, such as health and essential services workers, the adult work force, and adult females (to keep families together and take care of orphans) for postepidemic recovery.

It is self-evident to us that dose-sparing (diluting the vaccine) and postponing the next round of vaccinations (until more vaccine is produced), are the best solutions to the YF vaccine shortage. Even the 17 million doses WHO projects to be produced over the next few months will not fill current demand. From an abundance of caution, we recommend authorizing the use of a one fifth dose (*12*), before any more of the inadequate stocks are irrevocably depleted. WHO has indicated its approval to use such fractional dosage; application of this plan should be instituted immediately.
